# Editorial Correspondence

**Published:** 1876-07

**Authors:** 


### Editorial Correspondence.

### AMERICAN GYNECOLOGICAL SOCIETY.

It is about a year since Dr. James R. Chadwick, of Boston, conceived the idea of forming an American Gynecological Society, the object of which should be the promotion of knowledge in all that relates to diseases of women and to obstetrics.

After consulting personally and by letter with a number of the leading gynecologists of the United States, Dr. Chadwick called a meeting to be held on the 3rd of June, 1876, at the rooms of the Academy of Medicine, No. 12 West 31st Street, New York City.

The call was made by circular letters directed to such gentlemen as it was thought would be sufficiently interested to give their time and attention to the subject. In response to this call, between twenty and thirty prominent practitioners assembled, among whom were Drs. Peasley, Barker, Thomas, Lusk, Skeen, Trask, Mundé, Sims, Byrne, Noegerath, Goodell, Chadwick, Sinclair, Jenks, Parvin, Bixby, Taylor, Byford, etc.

The meeting was called to order by Dr. Chadwick, who moved that Dr. Peasley act as temporary chairman. The motion prevailed, and in taking the chair, Dr. P. stated the object of the meeting in a short but very appropriate address.

Dr. Chadwick was elected secretary *pro tem.*

On motion, the chairman appointed a committee, consisting of Drs. Thomas, Byrne and Parvin, to report a constitution and by-laws for the government of the society.

Dr. Barker entertained the gentlemen present by some remarks in reference to the objects and benefits of the organization until the committee entered with the report. The constitution and by-laws as reported by the committee—after discussion, in which Drs. Barker, Noegerath,

Sims, Parvin, Goodell, Chadwick, and Byford, took part —with very slight modification, were adopted.

A committee was then appointed by the chair to nominate permanent officers.

Drs. Sinclair, Trask, Lusk, Noegerath and Jenks constituted the committee.

The committee retired, and in a short time reported the names of Dr. Fordyce Barker, of New York, for President; Drs. Washington L. Atlee, of Philadelphia, and Wm. H. Byford, of Chicago, for Vice-Presidents ; Dr. James R. Chadwick, of Boston, for Secretary ; and Dr. Paul F. Mundé, of New York, for Treasurer.

These officers are, ex-officio, members of the council. Drs. J. Marion Sims, of New York ; Lyman, of Boston ; Goodell, of Philadelphia ; and Parvin, of Indianapolis ; were nominated as the other members of the council.

The report was adopted. This completed the organization of the society, whereupon the temporary chairman vacated the chair in favor of the president elect.

The future terms of membership are, that the candidate's name shall be presented by some member of the society to the council with an acceptable paper from the former on some subject contemplated in the organization, and ten dollars as the annual contribution. If the report of the council is favorable, and the candidate receives two-thirds of the votes of the members, he shall be declared elected.

The first annual meeting of the society is to take place in New York City, Sept. 13th, 1876, at the rooms of the Academy of Medicine, at which time it will be determined when the regular meetings will be held.

After the adjournment of the society, the council was called together by the president to make arrangements for the first annual session. The council appointed Drs. Mundé, Lyman, Goodell and the secretary to procure papers and make such arrangements as are necessary for the accommodation of the society.

Several papers are already promised, and it is confidently expected that a sufficient number of contributions will be received to make the meeting one of great scientific interest.

Entire unanimity prevailed, and I think we may reasonably expect good results from the harmonious co-operation of the gynecological practitioners of this country.

The profession of New York complain of the effect of hard times upon their cash receipts. W. H. B.

---

The following letter has been received from Iowa :

Gents. Mangerers of the Medical Departments.

jents Sir.

I want to know if I can make A Rangements with you to come & visit your Colledge. I have A man that is A live & is all Rite & his Neck is Broken the Boan is cleair Dislocated & is cleair A Peat it does not set one the Rite Place By an Half inch & he cant hold his head up onely By Bracers. it has Ben Broken A Bout 21 years he is the onely man that is liven in the none world that has got his Neck Broken or Dislocated he is A Object of cherity. Which he is the onely Person one Reckord of the kind & I want to find out from yu how meny Studiance yu have their at yu school & I want yu tu see yu Studiants & see how much of A Purce they will give me tu Bring him their How much each caller will give me to bring him their & then he will let any of the mangers Exam his Neck & Explain it to the school Why he lives & how he lives I cant explain it But yu can. he is tolible harty his health is tolible good, at the Preasant time As soon As yu can convenity see the Scollars at school & yu can Asitain what they will Each of them will give A Peace & Board him & my Self for the time that yu all Keep us their I think that 24 Hours will be long A nuff at A Place.

So Pleas let me know your conclucion as Soon as Posible

